# On the Structure, Economy, and Diseases of the Ear

**Published:** 1842-04

**Authors:** 


Art. III.—*A Treatise on the Structure, Economy, and Diseases of the Ear.* By George Pilcher, Lecturer on Anatomy and Surgery, &c. Second Edition.—*London,* 1842. 8vo, pp. 334.

The first edition of this work, a prize essay, was published in 1838, and gave a general view of the Anatomy, Physiology, and Diseases of the Ear. The author, however, passed over, in a few words, that class of diseases of the nature of which the profession has been hitherto quite ignorant, which, in reality, form the very great majority of the cases of deafness, and for which no means of relief have as yet been pointed out. In the edition before us, the author has added another coloured plate of ears with polypi, which, like all the others, is poor in design and execution, and appears to us be entirely useless to the surgeon; he has corrected some of the inaccuracies which we formely pointed out (British and Foreign Medical Review, vol. viii. p. 75), and he has added ten pages by way of a "conclusion," but in which we regret to say he has not given a single practical observation, although in the time which has elapsed between the two editions, he seems to have had many opportunities of studying this impoitant and interesting subject. The following extract from the "conclusion" shows that the author agrees with the profession generally in considering that, with our present knowledge, the majority of the diseases of the ear is incurable. At least, we presume that this is his meaning; although, from the almost unexampled inaccuracy of the language, it is not easy to say positively what the meaning is.

"The allusion in this summary to the extent of surgical aid in acquired causes of deafness, though brief, is sufficient to point to the fact, that in the present state of knowledge it is, to a distressing degree, unavailable, and that to a certain extent it will ever remain so; yet the daily advance of science justifies the hope that more may be achieved in this department also for suffering humanity." (p. 382.)

It will be naturally enquired, considering "the daily advance of science," why Mr. Pilcher has not added a single fact to this work since 1838, which can aid in the relief of "suffering humanity?" We very much regret that he should have been induced to publish another edition of his work without having done so, and he appears to us to be particularly open to censure for making no reference to the important researches of Mr. Wharton Jones and Mr. Joseph Toynbee; the former published in the Cyclopædia of Surgery, the latter in the twenty-fourth volume of the Medico-Chirurgical Transactions. These must be considered as the first attempts to discover the causes of the diseases of the ear; and the numerous dissections of Mr. Toynbee point out the most frequent seat of disease. How long will it be before surgeons cease to produce works which can be of no benefit either to the profession or to the public? In conclusion, we must, in justice to ourselves and to our readers, declare that this work is neither creditable to the author nor to British surgery; and that it still leaves the domain of aural pathology and therapeutics open to the unrestrained inroads of the charlatans who have so long disgraced it.

